# 2,6-Dibromo-4-formyl­phenyl 3-phenyl­prop-2-enoate

**DOI:** 10.1107/S1600536812037427

**Published:** 2012-09-08

**Authors:** C. Suresh Kumar, G. Jagadeesan, S. Dhamodaran, Karthik Ananth, S. Aravindhan

**Affiliations:** a Asthagiri Herbal Research Foundation, Perungudi, Chennai 600 096, India; b Department of Physics, Presidency College, Chennai 600 005, India

## Abstract

Mol­ecules of the title compound, C_16_H_10_Br_2_O_3_, adopt an *E* conformation about the C=C double bond. The dihedral angle between the two aromatic rings is 78.0 (7)°. In the crystal, mol­ecules are linked through weak C—H⋯O hydrogen bonds.

## Related literature

For the biological activity of cinnamoyl derivatives, see: De *et al.* (2011 ▶); Obioran *et al.* (1986 ▶); Cremlyn *et al.* (1984 ▶).
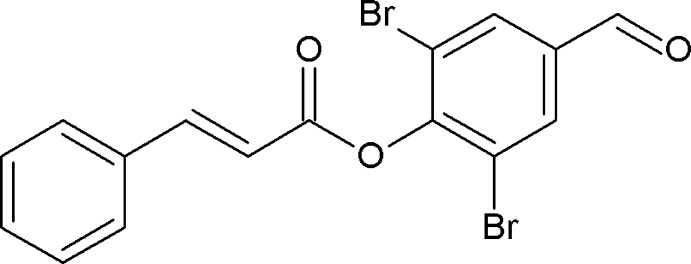


## Experimental

### 

#### Crystal data

C_16_H_10_Br_2_O_3_
*M* *_r_* = 410.06
Triclinic, 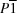

*a* = 8.0846 (3) Å
*b* = 9.0149 (4) Å
*c* = 11.8995 (5) Å
α = 77.429 (2)°
β = 73.918 (2)°
γ = 70.236 (2)°
*V* = 776.83 (6) Å^3^
*Z* = 2
Mo *K*α radiation
μ = 5.22 mm^−1^
*T* = 293 K
0.25 × 0.20 × 0.20 mm

#### Data collection

Bruker Kappa APEXII CCD diffractometer
Absorption correction: multi-scan (*SADABS*; Bruker 2004 ▶) *T*_min_ = 0.979, *T*_max_ = 0.983
15811 measured reflections
3764 independent reflections
2282 reflections with *I* > 2σ(*I*)
*R*_int_ = 0.029

#### Refinement

*R*[*F* ^2^ > 2σ(*F* ^2^)] = 0.041
*wR*(*F* ^2^) = 0.107
*S* = 0.98
3764 reflections
190 parameters
H-atom parameters constrained
Δρ_max_ = 0.63 e Å^−3^
Δρ_min_ = −0.52 e Å^−3^

### 

Data collection: *APEX2* (Bruker, 2004 ▶); cell refinement: *APEX2* and *SAINT* (Bruker, 2004 ▶); data reduction: *SAINT* and *XPREP* (Bruker, 2004 ▶); program(s) used to solve structure: *SHELXS97* (Sheldrick, 2008 ▶); program(s) used to refine structure: *SHELXL97* (Sheldrick, 2008 ▶); molecular graphics: *ORTEP-3 for Windows* (Farrugia, 1997 ▶); software used to prepare material for publication: *PLATON* (Spek, 2009 ▶).

## Supplementary Material

Crystal structure: contains datablock(s) I, global. DOI: 10.1107/S1600536812037427/bt6828sup1.cif

Structure factors: contains datablock(s) I. DOI: 10.1107/S1600536812037427/bt6828Isup2.hkl

Supplementary material file. DOI: 10.1107/S1600536812037427/bt6828Isup3.cml

Additional supplementary materials:  crystallographic information; 3D view; checkCIF report

## Figures and Tables

**Table 1:** Hydrogen-bond geometry (Å, °)

*D*—H⋯*A*	*D*—H	H⋯*A*	*D*⋯*A*	*D*—H⋯*A*
C5—H5⋯O1^i^	0.93	2.48	3.221 (6)	136

Symmetry code: (i) 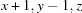.

**Fig. 1.**
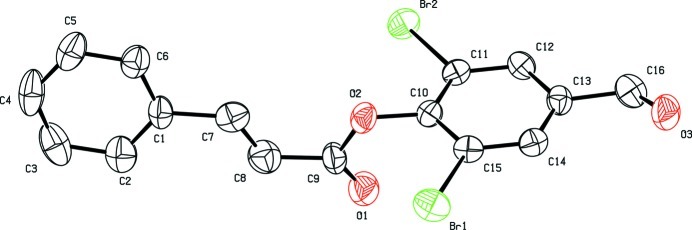
The molecular structure of the title compound showing 30% probability displacement ellipsoids. H atoms are omitted for clarity.

**Fig. 2.**
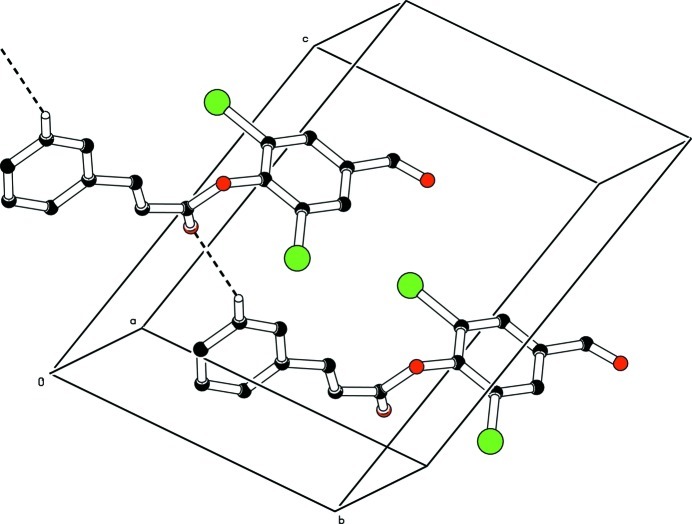
Crystal packing diagram. H atoms not involved in intermolecular hydrogen bonding (dashed lines) have been omitted for clarity.

## supplementary crystallographic information

### Comment

Cinnamoyl derivatives exhibit a variety of pharmacological properties, e.g anticancer, antitumor and antimicrobial activities (De *et al.*, 2011; Obioran *et al.*, 1986; Cremlyn *et al.*, 1984). In view of their importance, the crystal structure determination of the title compound was carried out and the results are presented herein. The molecular structure of the title compound is shown in Fig. 1. In the molecule, the configuration about the C═C double bond is *E*. The dihedral angle between the two aromatic rings is 78.0 (7)°. In the crystal packing, molecules are linked through weak C—H···O hydrogen bonds (Fig. 2).

### Experimental

To a solution of 3,5-dibromo benzaldehyde (0.03 mol) in chloroform (100 ml) cinnamoyl chloride (0.03 mol) was added followed by addition of triethyl amine (0.03 mol). Then the reaction was stirred at room temperature for 3 h. The reaction mixture was quenched with water and the chloroform layer was separated. The combined chloroform layers were washed with 5% NaOH solution followed by water wash and dried with sodium sulfate. Then the mixture was concentrated under reduced pressure. The obtained solid was crystallized in a mixture of methanol:chloroform.

### Refinement

H atoms were refined with fixed individual displacement parameters [U(H) = 1.2 *U*_eq_(C)] using a riding model with C—H = 0.93 Å.

### Crystal data

**Table:**

C_16_H_10_Br_2_O_3_	*Z* = 2
*M**_r_* = 410.06	*F*(000) = 400
Triclinic, *P*1	*D*_x_ = 1.753 Mg m^−^^3^
Hall symbol: -P 1	Mo *K*α radiation, λ = 0.71073 Å
*a* = 8.0846 (3) Å	Cell parameters from 8834 reflections
*b* = 9.0149 (4) Å	θ = 2.1–31.2°
*c* = 11.8995 (5) Å	µ = 5.22 mm^−^^1^
α = 77.429 (2)°	*T* = 293 K
β = 73.918 (2)°	Block, colourless
γ = 70.236 (2)°	0.25 × 0.20 × 0.20 mm
*V* = 776.83 (6) Å^3^	

### Data collection

**Table:**

Bruker Kappa APEXII CCD diffractometer	3764 independent reflections
Radiation source: fine-focus sealed tube	2282 reflections with *I* > 2σ(*I*)
Graphite monochromator	*R*_int_ = 0.029
ω and φ scan	θ_max_ = 28.2°, θ_min_ = 2.4°
Absorption correction: multi-scan (*SADABS*; Bruker 2004)	*h* = −10→10
*T*_min_ = 0.979, *T*_max_ = 0.983	*k* = −11→11
15811 measured reflections	*l* = −15→15

### Refinement

**Table:**

Refinement on *F*^2^	Primary atom site location: structure-invariant direct methods
Least-squares matrix: full	Secondary atom site location: difference Fourier map
*R*[*F*^2^ > 2σ(*F*^2^)] = 0.041	Hydrogen site location: inferred from neighbouring sites
*wR*(*F*^2^) = 0.107	H-atom parameters constrained
*S* = 0.98	*w* = 1/[σ^2^(*F*_o_^2^) + (0.0417*P*)^2^ + 0.7467*P*] where *P* = (*F*_o_^2^ + 2*F*_c_^2^)/3
3764 reflections	(Δ/σ)_max_ = 0.001
190 parameters	Δρ_max_ = 0.63 e Å^−^^3^
0 restraints	Δρ_min_ = −0.52 e Å^−^^3^

### Special details

**Table:**

Geometry. All e.s.d.'s (except the e.s.d. in the dihedral angle between two l.s. planes) are estimated using the full covariance matrix. The cell e.s.d.'s are taken into account individually in the estimation of e.s.d.'s in distances, angles and torsion angles; correlations between e.s.d.'s in cell parameters are only used when they are defined by crystal symmetry. An approximate (isotropic) treatment of cell e.s.d.'s is used for estimating e.s.d.'s involving l.s. planes.
Refinement. Refinement of *F*^2^ against ALL reflections. The weighted *R*-factor *wR* and goodness of fit *S* are based on *F*^2^, conventional *R*-factors *R* are based on *F*, with *F* set to zero for negative *F*^2^. The threshold expression of *F*^2^ > σ(*F*^2^) is used only for calculating *R*-factors(gt) *etc*. and is not relevant to the choice of reflections for refinement. *R*-factors based on *F*^2^ are statistically about twice as large as those based on *F*, and *R*- factors based on ALL data will be even larger.

### Fractional atomic coordinates and isotropic or equivalent isotropic displacement parameters (Å^2^)

**Table:**

	*x*	*y*	*z*	*U*_iso_*/*U*_eq_	
O2	0.5056 (3)	0.8407 (3)	0.3049 (2)	0.0643 (7)	
Br2	0.28505 (7)	0.69030 (5)	0.52006 (4)	0.07858 (18)	
Br1	0.54158 (7)	1.17400 (6)	0.21211 (4)	0.08435 (18)	
C10	0.3929 (4)	0.9517 (4)	0.3796 (3)	0.0499 (8)	
C15	0.3931 (5)	1.1088 (4)	0.3529 (3)	0.0555 (9)	
C12	0.1796 (5)	1.0109 (4)	0.5601 (3)	0.0562 (9)	
H12	0.1060	0.9786	0.6297	0.067*	
C1	0.8079 (5)	0.4509 (4)	0.1110 (4)	0.0612 (10)	
C16	0.0729 (6)	1.2807 (6)	0.6205 (4)	0.0745 (12)	
H16	−0.0078	1.2465	0.6849	0.089*	
C13	0.1840 (5)	1.1664 (4)	0.5332 (3)	0.0559 (9)	
C5	1.0667 (7)	0.2245 (5)	0.1043 (5)	0.0881 (14)	
H5	1.1658	0.1610	0.1351	0.106*	
O3	0.0815 (5)	1.4077 (4)	0.6125 (3)	0.0920 (10)	
C11	0.2853 (5)	0.9037 (4)	0.4828 (3)	0.0517 (8)	
C2	0.7771 (6)	0.4158 (6)	0.0153 (4)	0.0796 (13)	
H2	0.6803	0.4821	−0.0168	0.096*	
C9	0.4246 (7)	0.8097 (5)	0.2269 (4)	0.0657 (11)	
C7	0.6899 (6)	0.5893 (5)	0.1768 (4)	0.0704 (11)	
H7	0.7306	0.6079	0.2368	0.085*	
C14	0.2892 (5)	1.2157 (4)	0.4296 (3)	0.0573 (9)	
H14	0.2901	1.3212	0.4114	0.069*	
C8	0.5420 (7)	0.6811 (6)	0.1574 (4)	0.0830 (13)	
H8	0.5037	0.6671	0.0946	0.100*	
C6	0.9537 (6)	0.3553 (5)	0.1536 (4)	0.0721 (11)	
H6	0.9770	0.3801	0.2184	0.087*	
O1	0.2767 (5)	0.8782 (4)	0.2205 (3)	0.0886 (9)	
C4	1.0338 (8)	0.1871 (6)	0.0099 (6)	0.0954 (18)	
H4	1.1101	0.0970	−0.0237	0.114*	
C3	0.8902 (8)	0.2802 (7)	−0.0361 (5)	0.0943 (17)	
H3	0.8673	0.2543	−0.1006	0.113*	

### Atomic displacement parameters (Å^2^)

**Table:**

	*U*^11^	*U*^22^	*U*^33^	*U*^12^	*U*^13^	*U*^23^
O2	0.0470 (14)	0.0607 (15)	0.0710 (17)	−0.0052 (12)	−0.0006 (13)	−0.0138 (13)
Br2	0.1027 (4)	0.0525 (2)	0.0777 (3)	−0.0311 (2)	−0.0149 (2)	0.00322 (19)
Br1	0.0860 (3)	0.0953 (3)	0.0707 (3)	−0.0500 (3)	−0.0005 (2)	0.0062 (2)
C10	0.0397 (19)	0.0526 (19)	0.056 (2)	−0.0126 (15)	−0.0095 (16)	−0.0082 (17)
C15	0.050 (2)	0.063 (2)	0.056 (2)	−0.0249 (17)	−0.0117 (17)	−0.0009 (18)
C12	0.053 (2)	0.066 (2)	0.051 (2)	−0.0247 (18)	−0.0061 (17)	−0.0090 (18)
C1	0.064 (3)	0.052 (2)	0.062 (2)	−0.0278 (19)	0.0140 (19)	−0.0159 (18)
C16	0.072 (3)	0.090 (3)	0.074 (3)	−0.031 (2)	−0.016 (2)	−0.025 (2)
C13	0.052 (2)	0.059 (2)	0.063 (2)	−0.0162 (17)	−0.0154 (18)	−0.0196 (18)
C5	0.077 (3)	0.055 (2)	0.121 (4)	−0.017 (2)	−0.003 (3)	−0.016 (3)
O3	0.110 (3)	0.076 (2)	0.098 (2)	−0.0113 (19)	−0.037 (2)	−0.0376 (19)
C11	0.051 (2)	0.0473 (18)	0.060 (2)	−0.0200 (16)	−0.0151 (17)	−0.0012 (16)
C2	0.067 (3)	0.084 (3)	0.086 (3)	−0.033 (2)	0.005 (2)	−0.018 (3)
C9	0.079 (3)	0.057 (2)	0.060 (2)	−0.020 (2)	−0.002 (2)	−0.0219 (19)
C7	0.064 (3)	0.073 (3)	0.073 (3)	−0.034 (2)	−0.007 (2)	0.006 (2)
C14	0.061 (2)	0.052 (2)	0.067 (2)	−0.0254 (18)	−0.0200 (19)	−0.0054 (18)
C8	0.085 (3)	0.089 (3)	0.084 (3)	−0.029 (3)	−0.025 (3)	−0.017 (3)
C6	0.071 (3)	0.058 (2)	0.080 (3)	−0.022 (2)	−0.001 (2)	−0.009 (2)
O1	0.090 (2)	0.087 (2)	0.092 (2)	−0.0086 (19)	−0.0341 (19)	−0.0304 (18)
C4	0.083 (4)	0.067 (3)	0.131 (5)	−0.037 (3)	0.029 (3)	−0.047 (3)
C3	0.102 (4)	0.118 (4)	0.085 (3)	−0.069 (4)	0.015 (3)	−0.045 (3)

### Geometric parameters (Å, º)

**Table:**

O2—C9	1.393 (5)	C13—C14	1.370 (5)
O2—C10	1.394 (4)	C5—C4	1.355 (8)
Br2—C11	1.878 (3)	C5—C6	1.358 (6)
Br1—C15	1.878 (4)	C5—H5	0.9300
C10—C11	1.373 (5)	C2—C3	1.401 (7)
C10—C15	1.383 (5)	C2—H2	0.9300
C15—C14	1.371 (5)	C9—O1	1.161 (5)
C12—C13	1.379 (5)	C9—C8	1.473 (6)
C12—C11	1.379 (5)	C7—C8	1.252 (6)
C12—H12	0.9300	C7—H7	0.9300
C1—C2	1.352 (6)	C14—H14	0.9300
C1—C6	1.357 (6)	C8—H8	0.9300
C1—C7	1.512 (6)	C6—H6	0.9300
C16—O3	1.152 (5)	C4—C3	1.358 (8)
C16—C13	1.507 (6)	C4—H4	0.9300
C16—H16	0.9300	C3—H3	0.9300
			
C9—O2—C10	115.2 (3)	C1—C2—C3	120.4 (5)
C11—C10—C15	119.6 (3)	C1—C2—H2	119.8
C11—C10—O2	119.8 (3)	C3—C2—H2	119.8
C15—C10—O2	120.6 (3)	O1—C9—O2	122.1 (3)
C14—C15—C10	120.5 (3)	O1—C9—C8	124.1 (4)
C14—C15—Br1	120.0 (3)	O2—C9—C8	113.8 (4)
C10—C15—Br1	119.5 (3)	C8—C7—C1	125.6 (5)
C13—C12—C11	119.5 (3)	C8—C7—H7	117.2
C13—C12—H12	120.3	C1—C7—H7	117.2
C11—C12—H12	120.3	C15—C14—C13	119.6 (3)
C2—C1—C6	118.6 (4)	C15—C14—H14	120.2
C2—C1—C7	124.8 (4)	C13—C14—H14	120.2
C6—C1—C7	116.5 (4)	C7—C8—C9	124.6 (5)
O3—C16—C13	124.2 (5)	C7—C8—H8	117.7
O3—C16—H16	117.9	C9—C8—H8	117.7
C13—C16—H16	117.9	C1—C6—C5	122.0 (5)
C14—C13—C12	120.6 (3)	C1—C6—H6	119.0
C14—C13—C16	120.5 (4)	C5—C6—H6	119.0
C12—C13—C16	118.8 (4)	C3—C4—C5	120.5 (5)
C4—C5—C6	119.5 (5)	C3—C4—H4	119.8
C4—C5—H5	120.3	C5—C4—H4	119.8
C6—C5—H5	120.3	C4—C3—C2	119.0 (5)
C10—C11—C12	120.3 (3)	C4—C3—H3	120.5
C10—C11—Br2	119.8 (3)	C2—C3—H3	120.5
C12—C11—Br2	119.9 (3)		

### Hydrogen-bond geometry (Å, º)

**Table:**

*D*—H···*A*	*D*—H	H···*A*	*D*···*A*	*D*—H···*A*
C5—H5···O1^i^	0.93	2.48	3.221 (6)	136
C7—H7···O2	0.93	2.39	2.764 (3)	104

Symmetry code: (i) *x*+1, *y*−1, *z*.

## References

[bb1] Bruker (2004). *APEX2*, *SAINT* and *XPREP* Bruker AXS Inc., Madison, Wisconsin, USA.

[bb2] Cremlyn, R. J., Thandi, K. & Wilson, R. (1984). *Indian J. Chem. Sect. B*, **23**, 94–96.

[bb3] De, P., Baltas, M. & Bedos-Belval, F. (2011). *Curr. Med. Chem.* **18**, 1672–1703. 10.2174/092986711795471347 21434850

[bb4] Farrugia, L. J. (1997). *J. Appl. Cryst.* **30**, 565.

[bb5] Obioran, O., Cremlyn, R. J. & Singh, G. (1986). *Indian J. Chem. Sect. B*, **25**, 559–561.

[bb6] Sheldrick, G. M. (2008). *Acta Cryst.* A**64**, 112–122. 10.1107/S0108767307043930 18156677

[bb7] Spek, A. L. (2009). *Acta Cryst.* D**65**, 148–155. 10.1107/S090744490804362X PMC2631630 19171970

